# Fast-Speech-Induced Hypoarticulation Does Not Considerably Affect the Diachronic Reversal of Complementary Length in Central Bavarian

**DOI:** 10.1177/00238309221127641

**Published:** 2022-11-28

**Authors:** Markus Jochim, Felicitas Kleber

**Affiliations:** Institut für Phonetik und Sprachverarbeitung, Ludwig-Maximilians-Universität München, Germany

**Keywords:** Sound change, internal versus external, dialect leveling, speech rate, Bavarian

## Abstract

This study investigated a sound change in progress by which the Central Bavarian dialect feature of complementary length between consonant and the preceding vowel is giving way to the unrestricted combination possibility of long (Vː) and short (V) vowels with following longer fortis (Cː) and shorter lenis (C) stops, respectively. This 2 × 2 system is also found in the standard variety of German. While previous studies have regarded any such findings of convergence toward Standard German as being a result of language contact, the present study specifically tested the possibility of fast-speech-induced hypoarticulation being a system-internal driver of this change. The focus of this study was on acoustic cues to the postvocalic stop. Following the apparent-time paradigm, acoustic analyses of 10 younger and 10 older dialect speakers revealed that (1) younger dialect speakers produced both VC and VːCː (both formerly illegal in the dialect), but (2) older dialect speakers produced only VːCː sequences with duration patterns similar to those of the control group of 10 Standard German speakers. Analyses of various dependent variables further showed (3) the (apparently) delayed emergence of aspiration as an additional cue to the fortis–lenis contrast in Western Central Bavarian particularly in younger dialect speakers, (4) no considerable effect of speech rate on the dispersion of and overlap between any of the four vowel-plus-stop combinations, and (5) the irregular spread of this change that appears to be gradual. As such, the findings support a model of linguistic change that also accounts for gradual changes in dialect borrowing.

## 1 Introduction

The focus of this paper is on the trigger and the implementation of a prosodic sound change currently in progress in the German dialect of Western Central Bavarian (WCB). Recent work suggests that some dialect features are in the process of being dropped in favor of features that resemble Standard German more closely. This includes the phonetic implementation of stops and the phonotactics of word-medial vowel-plus-stop sequences. One aim is to investigate the potential role of dialect-internal factors that may also be involved in this presumably externally fostered change. Another aim is to work out the specifics of how the change is progressing in the dialect, that is, to what extent the different cues are affected.

At the center of the sound change that we are interested in is the implementation of word-medial post-vocalic stops. Standard German has a phonemic length contrast in vowels (/miːtә/ “rent” vs. /mItә/ “center”) and a phonemic fortis–lenis contrast in stops (/miːtәn/ “to rent” vs. /miːdәn/ “(they) avoided”) (Wiese, 1996). Fortis stops have longer closure phases (in word-medial position) and a higher voice onset time (VOT) than lenis stops (Jessen, 1998). Phonotactically, Standard German does not restrict the combination of vowels and stops: Both fortis and lenis stops can follow after either phonemically long or short vowels. That is, in addition to the examples above representing combinations of short vowel plus fortis stop, long vowel plus fortis stop, and long vowel plus lenis stop, respectively, Standard German also allows the combination of short vowel plus lenis stop as in /vIdɐ/ (“ram”) (adding up to a total of four possible combinations). However, Central Bavarian (CB), spoken in the south east of Germany (Western CB, hereafter WCB) and most parts of Austria (Eastern CB, hereafter ECB), puts a clear restriction on the combination of sounds: Long vowels only occur before lenis stops and short vowels only before fortis stops. Most accounts claim for CB varieties that it is the stop contrast that is phonemic, while vowel length is regarded allophonic, that is, predictable by the nature of the post-vocalic stop (see, for example, Seiler, 2005; Wiesinger, 1990). As opposed to Standard German, the CB fortis–lenis contrast is a true length contrast (with fortis meaning long and lenis meaning short), which is why the phonotactic rule described above is often called one of complementary length (Hinderling, 1980). VOT plays much less of a role in the dialect (Bannert, 1976; Seiler, 2005; Wiesinger, 1990; but see Luef (2020) for VOT becoming more important in prevocalic stops in ECB dialects).

The rule of complementary length has been described in the literature for a long time (e.g., Bannert, 1976; Hinderling, 1980; Pfalz, 1913; Seiler, 2005; Wiesinger, 1990, where it has variously gone by the names of “Pfalz’s law,” “complementary length,” or “(Central) Bavarian quantity relations”). Hinderling (1980) leaves no doubt that the rule is adhered to even when borrowing words from Standard German into the dialect. According to him, borrowers avoid illegal combinations by adjusting either the vowel or the consonant. This is done in free variation. That is, a word such as *Pudding* “pudding”—a loan from Standard German,^1^ where it is produced as / pʊdɪŋ /—becomes either /puːdɪŋ/ or / pʊtɪŋ / in CB. But the same can also be observed in words native to the dialect lexicon, such as *Vater* “father,” /faːtɐ/ in Standard German, which is produced in the dialect either as /fatɐ/ (i.e., with a short vowel plus fortis stop) or as /fɔːdɐ/ (i.e., with a long vowel plus lenis stop).

While the literature on CB was unanimous about the above-mentioned phonotactic restriction for a long time, recent work suggests that the co-dependency of vowel and following stop is likely on the retreat, giving way to two independent phonemic contrasts between vowel length and consonant strength (i.e., fortis vs. lenis), respectively. Based on auditory analyses in the framework of traditional linguistic fieldwork, Schikowski (2009, p. 44f.) notes that the co-dependency can be weakened in younger speakers, although adherence to the rule is still absolutely dominant. Moosmüller and Brandstätter (2014) and Klingler et al. (2017) presented acoustic evidence that a combination of long vowel plus fortis consonant does form part of ECB, in spite of traditional accounts. Two further studies presented experimental evidence for more pronounced dialectal traces in older compared with younger WCB speakers’ production and perception of the Standard German vowel length (Kleber, 2020) and fortis–lenis contrast (Kleber, 2018), respectively. More precisely, older WCB speakers use consonant duration in both modalities to a greater extent than younger WCB speakers to cue the Standard German vowel length contrast before fortis stops in this regionally accented standard register. Moreover, only older WCB speakers adopted this strategy to differentiate vowel length in the context of post-vocalic sonorants. Regarding the fortis–lenis contrast, in comparison with the older cohort of the same speech community, younger WCB speakers were less affected by the prevalent dialectal complementary length feature again when asked to operate in a standard register and in particular in perception. At the same time, these younger speakers relied to a greater extent on VOT. Although all of these observations stem from sources that used very different methodologies and materials, they lend strong support for an acoustic apparent-time investigation of how WCB speakers from two different generations nowadays implement the postvocalic fortis–lenis contrast when operating in the dialect. The present study also fills the gap regarding the status of short vowel plus lenis stop combination (which to our knowledge has not been investigated before) by including this sequence in the acoustic analyses.

In investigating such vowel-plus-stop sequences, several phonetic studies (including those in the previous paragraph) have used combined measures like the vowel-to-closure duration ratio to describe the entire sequence. This has been claimed to be perceptually relevant (Kohler, 1979), more stable across speech styles (Pickett et al., 1999), or advantageous for typological reasons (Jochim & Kleber, 2017). The present study, however, is largely built on the separate measure of closure duration to allow for a more detailed tracking of the change within the two speech sounds.

Kleber (2020) argued that dialect leveling, which is defined as a diachronic process during which regional varieties become more similar to the standard language (Kerswill, 2003; Trudgill, 1986) or a close dialect (Hinskens, 1998) as a result of language-external factors such as changing community network structures and speaker mobility (Britain, 2010), accounts best for this observed change. Instead of continuing to use the term of leveling—which is less specific about the direction of change—we will use in this paper the term convergence to specifically describe the convergence toward the standard.

Although this process of convergence (or leveling for that matter) has been related to a number of other sound changes currently in progress in Germany (cf. Harrington et al., 2012 and Bukmaier et al., 2014) and other European countries (e.g., Kerswill, 2002 on British English; Hewlett et al., 1999; Scobbie, 2005 on Scottish English), the possibility for this change to be triggered by system-internal phonetic factors such as speech-rate induced hypoarticulation (see below) has hardly been considered in these studies. Some of them focused on the process of gradual changes in progress in perception and production and only subsequently discussed potential triggers for the spread of the change (e.g., Bukmaier et al., 2014; Harrington et al., 2012, but see Kleber, 2020 for a longer discussion of internal factors); others investigated sociolinguistic factors involved in the spread of a change (Eckert, 2012; Labov, 2001). The vast majority of sociophonetics or sociolinguistic studies on sound changes in progress are—by their very nature—on the spread of a change across generations and speaker communities, whereas historical linguistics and phonetic models of sound change most often deal with the actuation of a change (see Baker et al., 2011; Ohala, 1993a), be it retrospectively after the completion of a then obvious and categorical change (e.g., Iverson & Salmons, 2012) or in the laboratory to better understand the mechanisms of a change’s origin based on synchronic data (e.g., Ohala, 1990). The present study investigates for the first time in the laboratory whether internal factors play a role in a presumed sound change in progress that has been related previously to external factors only. In other words, and through reversing a quote by Blust (2005) who questioned whether sound changes must be linguistically motivated, we put to the test whether an apparently externally motivated spread of a sound change may in fact also be driven by internal factors.

This is important for various reasons: (1) internal and external factors are very likely intertwined in sound change processes, as accumulating evidence for a greater complexity of type and spread of sound change suggests (see below and Hansen, 2001; Kerswill, 2002; Phillips, 2015). (2) Other (prosodic) changes regarding quantity have been proposed to be conditioned by phonetic and or phonological factors, for example, lenition of fortis stops (Hualde et al., 2011), open syllable lengthening in Middle High German, Middle Dutch, and Middle English (Lahiri & Dresher, 1999) or the emergence of the Estonian three-way quantity contrast in vowels and intervocalic consonants in disyllables (Lehiste, 2011). (3) A recent study by Rathcke and Stuart-Smith (2016) has in fact presented phonetic evidence that an ongoing prosodic change in Glaswegian English, which—similarly to the Bavarian case—affects the phonotactic restriction of vowel length and which has been related to convergence in earlier studies (Hewlett et al., 1999; Scobbie, 2005), is more likely related to a language-internal factor (here hypoarticulation due to prosodic deaccentuation) than to language contact.

The language-internal factor chosen for the present investigation is that of speech rate-induced hypoarticulation, which appears especially relevant to the collapse of duration-based contrasts, and which appears in everyday speech. More precisely, hypoarticulation arising from fast speech constitutes a phonetic bias (Garrett & Johnson, 2013) able to trigger a change toward short vowels and lenis consonants. This builds on ideas from Kohler (1984) and Ohala’s (1993a) model of listener errors leading to sound change. The model differentiates how listeners usually handle phonetic variance from an unusual, erroneous way that can give rise to sound change but is only observed infrequently. One source of phonetic variance is, for example, coarticulation, where a phonological property of one speech sound is physically present in another speech sound (Farnetani & Recasens, 2010). Usually, in Ohala’s model, listeners will compensate for this displacement and thus be able to attribute the property to the speech sound it originated from, rather than the speech sound it physically appeared in. In infrequent situations, however, listeners fail to achieve this compensation: They wrongly attribute the property to the speech sound it physically appears in and may eventually adjust their mental representation of the respective speech sounds (as happens, for instance, in tonogenesis, see Kingston, 2011).

Transferring this model to duration-based contrasts, we aim to test whether fast-speech-induced hypoarticulation should be regarded a potential system-internal factor for triggering the sound change in question—as it may lead eventually to failing compensation for hypoarticulation. Fast-speech-induced hypoarticulation is ubiquitous in everyday speech (Lindblom, 1990) and listeners are usually very well able to compensate for fast speech (Reinisch, 2016). However, fast speech can be a particular peril to phonologically long sounds that contrast with a short counterpart. Mitterer (2018) showed that in Maltese, “the singleton-geminate distinction is endangered by speech-rate variation” (p. 1). However, he also highlights cross-linguistic differences in the variation between singleton and geminate duration. This suggests that in some languages, speech rate variation may not be large enough to endanger the contrast and these languages might thus have no need to employ compensation for speech rate. This, in turn, would mean that only languages where speech rate variation is large enough in the first place are susceptible to the failure-of-compensation-based account of diachronic shortening outlined above, which prompts us to explore such effects of “endangerment” in the present study. Although Bukmaier and Harrington (2016) found no effect of fast-speech induced hypoarticulation on the rare three-way contrast /s, ʂ, ɕ/ in standard Polish, their findings may be related to the fact that they tested a variety that shows no signs of losing this contrast (despite the observation of such mergers in a number of Polish varieties). In the present study, we, therefore, will test again the effects of fast-speech-induced hypoarticulation on the phonetic implementation of phonemic contrasts, but with speakers of both stable (here the non-changing German standard variety) and unstable varieties (here WCB) and with different measures of a duration-based rather than spectrum-based contrast. The prediction is to find greater effects of speech rate in the form of greater within-category variability and between-category overlap in the speakers of the unstable variety than in speakers of the stable variety. No a priori predictions are made with respect to potential age group differences within the unstable variety; in this regard, the study is exploratory.

The three main aims of the present paper are therefore to test (1) whether long vowel plus fortis stop sequences emerge in the WCB dialect (as shown for ECB in Moosmüller and Brandstätter (2014) and suggested by WCB speakers’ usage of the regionally accented standard register (Kleber, 2018, 2020)), (2) whether this change extends to short vowel plus lenis stop sequences, and (3) whether such a change can also be related to language-internal factors (as was the case for Glaswegian English).

To test these predictions, we collected original recordings from both dialect speakers and standard speakers in a controlled speech production experiment using the apparent-time paradigm. The basic assumption underlying this paradigm is that diachronic changes in a linguistic system emerge as generational differences in synchronic comparisons of that system (Bailey et al., 1991), given that an adult speaker’s pronunciation remains stable once the process of first language (L1) acquisition has been completed in late adolescence (Sankoff & Blondeau, 2007). Hence, in the apparent-time construct, the speech of a 60-year-old participant is seen to represent the state of his L1 linguistic system 45*–*40 years ago. One variable we controlled for was speech rate, eliciting the speakers’ usual tempo as well as the highest tempo they would comfortably employ. The words elicited were the same for both varieties. This allowed us a direct comparison of dialect realizations with the standard variety as a control group. We constructed a number of dependent variables to analyze the WCB dialect and the suspected sound change not only at the phoneme level, but also at the word level.

The results complement and extend previous findings on the dialects and regional standards spoken in the CB dialect area (Bannert, 1976; Hinderling, 1980; Kisler & Kleber, 2019; Kleber, 2020; Moosmüller & Brandstätter, 2014; Pfalz, 1913; Schikowski, 2009; Seiler, 2005; Wiesinger, 1990). To our knowledge, this is the first study to present experimental findings on VC words (i.e., short vowel plus lenis consonant) in these varieties; and the first controlled experiment after Bannert that deals with dialect rather than regional standard data from the WCB area. This study adds new and important findings about the sound change in progress that was already suggested by Moosmüller and Brandstätter (2014), Kleber (2018, 2020) and to some extent also Schikowski (2009).

None of these studies explicitly relates this change to the emergence of the fortis–lenis contrast in syllable-initial, prevocalic position, although independent reports of such an emergence exist for the regional accent of WCB (Kleber, 2018) and ECB dialects (Luef, 2020). Despite such observations, it is important to analyze diachronic changes regarding the contrast for each position separately, and to be careful with generalizations: First, while the contrast has been considered merged in syllable initial position, the contrast always existed in post-vocalic position—albeit in co-dependency with the preceding vowel. Second, while the change in post-vocalic position seems to affect primarily closure duration and secondarily other cues, any change in syllable-initial position has to be primarily a change of VOT. For these reasons, we focus on the fortis–lenis contrast in post-vocalic position only.

Furthermore, the present study investigates the potential sound change in progress not only on the phoneme but also—though only preliminarily and by means of a descriptive analysis—on the word level to shed some light on the nature of this change. Externally driven changes such as dialect borrowing via contact are often considered to be phonetically categorical and abrupt (i.e., an instant change on the phoneme level) and to spread irregularly (see, for example, Labov, 1994); that is, different words are affected by the change at different points in time—a process that is also termed lexical diffusion. As this is a preliminary analysis, we follow here Crystal’s (2008, p. 145) rather general definition of lexical diffusion, treating it as a surface phenomenon by which different lexical items can be affected to different degrees. However, gradual changes in the fine phonetic implementation of a change that may only eventually lead to a change on the phoneme level are regarded to be driven by system-internal factors and to spread regularly; that is, all words containing the sound undergoing change are affected at the same time. Recent evidence, however, suggests that both external and internal factors may play a role in gradual sound changes (Torgersen & Kerswill, 2004) which can also spread irregularly (e.g., Hansen, 2001; Yaeger-Dror, 1996 for vocalic changes in varieties of French, and Krishnamurti [1998], for fricative lenition and weakening in Gondi dialects)—just as externally motivated changes as a result of dialect leveling may spread both regularly and irregularly (see Kerswill [2002] for examples of both in dialect leveling in British English).

## 2 Method

### 2.1 Participants

This analysis includes data from 30 speakers in three groups: 10 younger dialect speakers (aged 20*–*29 years, mean 25.3, standard deviation 2.91; 6 female, 4 male), 10 older dialect speakers (aged 49 years and above, mean 60.5, *SD* 8.15; 5f, 5m), 10 younger standard speakers (aged 19*–*30, mean 24.1, *SD* 3.51; 4f, 6m). The standard speakers were all from Munich and served as a control group. For the standard group, we only selected speakers who did not speak dialect according to their own assessment (they had varying degrees of passive knowledge of the dialect). Their variety of Standard German can be described as Southern Standard German.^2^ For the dialect group, we selected speakers from the WCB dialect region, mostly from the district of Upper Bavaria. Their dialect competence was assessed by the first author, a native speaker of WCB.

All of our dialect speakers—regardless of age—are bilingual in the sense that they regularly speak the dialect and are able to operate in some form of standard register.^3^ Among both age groups were (1) speakers with a high-school diploma (*Abitur/Fachabitur*; though this number was—following a general trend in Germany—slightly higher in the younger group) and (2) speakers who use the standard register more often than others. Although this form of bilingualism is likely to contribute to contact-induced change, all dialect speakers are representative regarding the status quo of WCB; our younger WCB speakers do not use the dialect less often compared with our older WCB speakers.

### 2.2 Materials

The analysis included the 25 trochaic two-syllable words listed in Table 1, which were taken from a larger corpus and which are part of both the Standard German and the CB lexicon. The selection of words for analysis were based on the following three criteria: All words

- Exist in both lexica (although one can be a native word in one lexicon and a loan in the other),
- Are part of a (near) minimal set,
- Are of the structure $C_{1}VC_{2}X$ , with $C_{1}$ being any consonant, $C_{2}$ being a stop, and V being a vowel. X was either one of the two vowels /ә/ or /ɐ/, or the sequence /әn/ (where speakers sometimes elided the Schwa), or the sequence /ɪŋ/ (the latter only in the word *Pudding*).

**Table 1.** Words used in the present study, grouped by their phoneme types in Standard German.

VːC		VːCː		VC		VCː	
wieder	4.613	Bieter	0.954	Widder	1.415	bitter	2.167
Puder	1.681	Pute	1.415	Pudding	2.179	Butter	2.430
Tube	1.556	Lupe	2.004			Suppe	2.731
Hagen	1.886	Haken	2.604			hacken	2.238
Kader	1.079	Kater	7.760			Cutter	1.756
Rabe	1.431			Rabbi	2.021	Rappe	0
Tiger	2.642			Tigger	0	Ticker	0.845
		bieten	2.851			bitten	3.334

*Note.* V denotes a short vowel, Vː a long vowel, C a lenis consonant, and Cː a fortis consonant. Every row is one minimal set or near-minimal set. The numbers indicate the lgSUBTLEX value (log lexical frequency per million) in the SUBTLEX corpus (Brysbaert et al., 2011).

The target sounds were V and $C_{2}$ . We focused on (near) minimal sets as they provide a reliable basis for the fine-phonetic analyses described in the present study. Although this limited our amount of speech material, an analysis of 25 words distributed across eight (near) minimal sets outnumbers any previous experimental phonetic analyses of the WCB dialect. We had to resort to near-minimal sets instead of minimal sets in some cases, because actual minimal sets meeting our criteria are not part of the lexicon. We made sure that the word-initial consonants shared the same place of articulation within each set. We have no reason to believe that problems for our analyses arise from the fact that some sets were only near-minimal.

To incorporate as many minimal sets as possible, the target stops include all three places of articulation (labial, alveolar, and velar), where Standard German and CB have stops; the target vowels include the long and short variants of an i-like, u-like, and a-like vowel. The variation in the quality of V and the place of articulation of $C_{2}$ was balanced across the four categories of interest as far as possible. The apparent higher number of words with alveolar stops is conditioned by the greater prevalence of alveolar fortis stops after long vowels. We decided to allow for this bias, as it is representative of the Standard German lexicon (e.g., Féry, 2003; Kleber et al., 2010) and because vowel quality and place of articulation were not included as predictor variables in the present study (see Section 2.6).

The variation in the lgSUBTLEX values in Table 1, each reflecting a word’s log frequency per million value in the SUBTLEX corpus (Brysbaert et al., 2011), was similar across words with VC and VːCː sequences (i.e., those that are phonotactically illegal in Bavarian), on one hand, and words with VːC and VCː sequences (phonotactically legal clusters in Bavarian), on the other hand. A Wilcoxon rank-sum test with continuity correction revealed no significant effect of the fixed factor dialectal phonotactic restriction (two levels: illegal vs. legal in Bavarian) on the dependent variable lgSUBTLEX value ( W=72.5,p=0.9116 ).^4^

The words were embedded in carrier sentences in a way that made it likely for them to carry the sentence accent. More precisely, each target word occurred before a phrase-final verb, with each verb having a high co-occurrence probability with the preceding target word (e.g., *Suppe kochen*, lit. “soup cook”). The number of words preceding the target words varied between two and three. The first word was always the subject (*Ich*, *Er*, *Sie*, *Das*, “I, He, She, That”), the second word was always an inflected modal or auxiliary verb (e.g., *kann*, *will*, “can, want”), and the optional third word, for example, an adverb or article (see Appendix for all carrier sentences and translations). This way, the target word can be considered new information and is likely to be produced with a pitch accent. The sentences varied per word, such that the sentence plus target word combination was meaningful. All 10 repetitions (five per condition, see Section 2.3) of a word were embedded into the same carrier sentence.

The dialect sentences were translations of the standard sentences and matched those in position of the target word and meaning. As we only used target words that exist both in the standard variety and the dialect, lexical frequency is considered similar across varieties; that is, Puder and Butter each represent an equally lower- and higher-frequency word in Standard German and the dialect.

While the standard speakers were presented with prompt texts in standard orthography, the dialect group was presented with carrier sentences written in a way that non-linguists are likely to use when writing dialectal text messages, using the letters of the standard language’s alphabet. However, to elicit natural productions of the target words, we had to minimize the effect of the exact way words were spelled.^5^ We, therefore, used standard orthography for the target words, but not the carrier sentences (e.g., WCB *Sie woit an Pudding kocha.* instead of the Standard German orthography *Sie wollte einen Pudding kochen.* “She wanted to cook pudding.”).^6^ Moreover, for both dialect and standard, we made the prompt texts disappear before participants started reading, such that they did not see the written words while talking. Dialect speakers but not standard speakers were given the full list of prompt sentences immediately before the experiment to familiarize themselves with the chosen spelling. None reported any trouble with reading the sentences, nor did they show disfluencies in production during the experiment—neither in the fast nor the normal speech rate condition.

### 2.3 Procedure

Speakers produced the sentences in alternating speech rate blocks. To determine speaker-specific sentence duration, we asked participants, in a test phase prior to the experiment, to read out six different sentences, three *at their usual speaking rate*, and three *as fast as they could while still feeling comfortable*.^7^ We then measured the length of these utterances and averaged the length per condition, rounding to a multiple of 100 ms. 400 ms were added to allow the participants some time for preparing to speak. This typically resulted in values between 1,000 and 2,000 ms for both conditions. The difference between the two conditions was 200 or 300 ms for 28 participants and 400 ms for the remaining two participants.

The main phase began with a normal speech rate block. Participants were given 1.5 s to read the sentences silently from a screen. The text was then replaced by a progress bar that visualized the predetermined speaker-specific sentence duration at a normal speech rate. They now had to reproduce the sentence aloud from their memory. The task was to utter the sentence during the time the progress bar completed. After all sentences were spoken that way, the block was over. In the next block, participants were presented with the same prompts again, but while the time to silently read the sentence remained the same as in the previous block, participants were given less time to produce the sentence. The time given in this fast speech block was again indicated by the progress bar—now set to the predetermined speaker-specific fast speech rate. This procedure of alternating speech rate blocks was repeated 10 times, resulting in five repetitions of each token at a normal speech rate and five repetitions at a fast speech rate. Speakers were informed about the targeted speech rate prior to each block. Prompts were presented in randomized order, with each block containing a different order of prompts. The randomization was the same for all participants.

The SpeechRecorder software (Draxler & Jänsch, 2004) was used to present prompts and make recordings. The acoustic recordings were conducted either in a sound-attenuated recording booth at the Institute of Phonetics and Speech Processing in Munich, or with mobile equipment in quiet environments in participants’ homes. In either case, a head-mounted Beyerdynamic Opus 54 condenser microphone was used. The audio signal was digitized at a sampling rate of 44.1 kHz and a resolution of 16 bit, using a PreSonus audio interface in the studio and an M-Audio device in the mobile setting.

### 2.4 Segmentation and measurements

**Figure 1.**
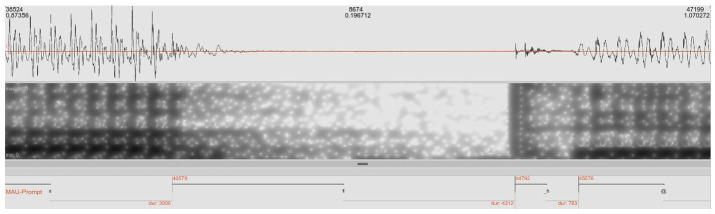
Segmentation example of a vowel-closure-aspiration-vowel sequence.

The complete utterances were automatically segmented with MAUS (Kisler et al., 2016) on three separate levels: utterance, word and phoneme level. Based on the automatic results, the relevant segment boundaries were checked and adjusted manually with the EMU Speech Database Management System (Jochim, 2017; Winkelmann et al., 2017). At this time point of data processing, we also checked auditorily whether the target word had been realized with a pitch accent. Only words that were accented on the phrase level were included in the analysis. The relevant segment boundaries were (1) the start and end of the utterance, (2) the start and end of each word, and (3) the start and end of each phoneme in the target word. The end of each segment was defined to coincide with the start of the respective following segment. The beginning and end of the two vowels of each target word were set to the center of the first and last visible vertical bar, respectively, in the spectrogram. This criterion was chosen to allow the best possible consistency across different labelers at the expense of possibly (but systematically) underestimating vowel duration and overestimating the duration of vowel-adjacent segments.^8^ These vowel boundaries, then, determined the boundaries of the adjacent segments, notably the start of the closure and the end of aspiration in $C_{2}$ (with $C_{2}$ being at the center of investigation in the present study). $C_{2}$ was then further subdivided into the **closure** and **aspiration phase** by adding manually an additional segment boundary at the point in time where the closure of the target word’s stop is released. More specifically, the burst was identified using the intensity spike in the wave form which is considered to mark the beginning of the **aspiration phase**. A segmentation example is given in Figure 1. The aspiration phase is thus identical to VOT, which was positive in most cases and never negative. In some cases the vertical bar of $V_{2}$ coincided with the burst of $C_{2}$ , for example, when dialect speakers realized lenis stops as approximants, which is a typical surface form of hypoarticulated lenis stops in CB. These instances were included in our analyses as stops with a VOT of 0.

### 2.5 Data analysis and dependent variables

A total of five dependent variables were then derived from the measurements described in Section 2.4. Before turning to the statistical models in Section 2.6, we will first describe the motivation for and the calculus behind each of these dependent variables that served different hypothesis testings.

#### 2.5.1 $closure_{norm}$ and $VOT_{norm}$

To tackle the question of a sound change in progress regarding stressed vowel-plus-stop sequences, we focused on *word-normalized closure duration*, henceforth $closure_{norm}$ , and *word-normalized voice onset time*, henceforth $VOT_{norm}$ , as our first two dependent variables.

**Definition 1**: $closure_{norm}$ of a given word token is defined here as the duration of the stop closure (i.e., from the offset of the preceding vowel to the burst) divided by target word duration.
**Definition 2**: $VOT_{norm}$ of a given word token is defined here as the duration of stop aspiration (i.e., from the burst to the onset of the following vowel) divided by target word duration.

We chose to word-normalize the segment durations instead of analyzing absolute duration values to account for token-specific general speech rate effects.

#### 2.5.2 Optimal category boundary

Following the method described in Miller et al. (1986) and Mitterer (2018), we then derived our third dependent variable, the *optimal category boundary* between fortis and lenis stops. It was used to test whether speech rate-induced variation in stop closure duration potentially endangers the fortis–lenis contrast.

**Definition 3**: The *optimal category boundary* for a given speaker at a given speech rate is defined here as the threshold value for stop closure duration that divides all stop tokens of one speaker and at the given speech rate most accurately into fortis and lenis stops.

To determine the threshold, we considered every integer value between the minimum closure duration of 0 and the maximum closure duration of 280 ms^9^ as a threshold candidate. Each threshold candidate divides all tokens of one speaker and at one speech rate into the fortis and lenis category, respectively, and for each candidate, the number of correctly classified tokens is calculated. The candidate with the highest number of correctly classified tokens was then selected to represent the optimal category boundary for the given speaker at the given speech rate.

We make the conjecture that large variation in the *optimal category boundary*—either between speakers or between speech rates—is indicative of instability in the fortis–lenis contrast. Small variation in *optimal category boundary*, however, would signify contrast stability.

#### 2.5.3 Dispersion difference

A second measure of a category’s (in-)stability within and across speakers and speech rates and our fourth dependent variable was the between-speech-rate difference in the dispersion of closure duration, henceforth *dispersion difference*. For its definition, we make use of the coefficient of variation (CoV), a standardized measure for dispersion that is defined as the ratio of the standard deviation of a measure to the mean of the same measure ( $\frac{StandardDeviation}{Mean}$ ).

**Definition 4**: The *dispersion difference* of a given speaker and a given word is defined here as the CoV of $closure_{norm}$ in fast speech minus the CoV of $closure_{norm}$ in normal-paced speech, with both CoV values calculated across all repetitions of the given word uttered by a given speaker at the respective speech rate.^10^

Positive *dispersion difference* values, therefore, indicate that $closure_{norm}$ is more variable in fast speech; negative values indicate that it is more variable in normal-paced speech; and 0 indicates that it is equally variable in both speech rates. Based on the assumption that only stable categories will absorb the presumed exertion of pressure in faster speech, while an unstable category should be adversely affected, we conjecture high values to be indicative of instability in the respective phonological category. More specifically, we predicted *dispersion difference* values around 0 for any category in the standard speakers assuming that all four categories are stable in this variety and, therefore, should not be affected by rate-dependent *dispersion differences*. If speech rate plays a role in the suspected sound change in progress regarding VC and VːCː sequences, then we expect positive values in at least one of the dialect groups and the two categories currently undergoing change. In other words, positive *dispersion difference* values may be seen as an indicator for the rate-induced instability of a phonological category.

#### 2.5.4 Fortis–lenis overlap

By means of a fifth dependent variable, we quantified again the possibility for category overlap in consequence of diachronic category instability, but this time from the perspective of potential word-specific effects.

**Definition 5**: For a given speaker, a given speech rate, and a given word including either a postvocalic fortis or lenis stop, the *fortis–lenis overlap* is defined here as:
  - The number of tokens of a particular word at a given speech rate that the speaker realized with a closure duration typical of the other stop category at the same speech rate,
  - divided by the total number of tokens of the word that the speaker realized at that speech rate.

More precisely, to calculate the speaker-specific *fortis–lenis overlap* for a given word with a fortis stop (e.g., *Kater*), we first averaged the closure duration across all repetitions of all words with lenis stops at the same place of articulation (i.e., *Kader, wieder*, etc. in this example) per speaker and speech rate and then computed the share of target fortis tokens (i.e., *Kater*) whose closure duration was below the third quartile of closure duration for lenis words. To calculate the speaker-specific *fortis–lenis overlap* of a given word with a lenis stop (e.g., *Rabbi*), we analogously averaged the closure duration across all repetitions of words with fortis stops at the same place of articulation (e.g., *Lupe, Suppe*) per speaker and speech rate, but computed the share of target lenis tokens (i.e., *Rabbi*) with a closure duration above the first quartile of closure duration for fortis words. The *fortis–lenis overlap* of a word can take values between 0 and 1, meaning no tokens or all tokens of the word, respectively, were produced in a way typical of the opposite kind of stop. A value of 1 (all tokens produced in a way typical of the opposite kind of stop) can of course only be expected in special circumstances. However, both of the two extreme values 0 and 1 would be indicative of a stable word category, whereas other values, especially those near 0.5, would be indicative of a word undergoing phonetic or phonological change.

### 2.6 Statistics

All statistical analyses were conducted with the statistical software R (version 3.6.1, R Core Team, 2019) and the R packages lmerTest (version 3.1-0, Kuznetsova et al., 2018), lme4 (version 1.1-21, Bates et al., 2019), and emmeans (version 1.4, Lenth et al., 2019).

For the analyses in Sections 3.1, 3.2, and 3.3, we fitted three linear mixed-effects models on our data. The main purpose of this was to be able to estimate marginal means for the various factors. This was not used for the word-by-word analysis in Section 3.4, because the measure used as a dependent variable there (fortis–lenis overlap, see Section 2.5) strongly reduces the raw data; this enables us to consider the entirety of fortis–lenis overlap data points in an analysis using visualization and basic descriptive tools (thus eliminating the need for estimating marginal means). A second reason for fitting linear mixed-effects models was to test the statistical significance of factors.^11^

Two linear mixed-effects models included the dependent variables word-normalized closure duration ( $closure_{norm}$ ) and word-normalized voice onset time ( $VOT_{norm}$ ), respectively. Both of them included the fixed factors *speaker group* (younger standard speakers, younger dialect speakers, older dialect speakers), *quantity category* (VːC, VːCː, VC, VCː, with V denoting a vowel, C a consonant, and ː being the length marker), and *speech rate* (fast, normal);^12^ and the random factors *speaker* and *target word*. The third model included the dependent variable *dispersion difference*; it included the same set of fixed and random factors as the other two models, with the exception, of course, that *speech rate* was not included (the dependent variable already incorporates both speech rates). The models were specified as shown in Equations 1, 2, and 3, respectively.^13^ In all three models, both random slopes and random intercepts were included as they are within factors with regard to each random factor.

Based on these models, we carried out pairwise comparisons using Tukey’s method for correcting family-wise errors.

(1) $$\begin{array}{l} closure\_{norm}^{\sim }speaker\_group*category*rate\\ +\;(category+rate|speaker)+(speaker\_group+rate|target\_word) \end{array}$$

(2) $$\begin{array}{l} vot\_{norm}^{\sim }speaker\_group*category*rate\\ +\;(category+rate|speaker)+(speaker\_group+rate|target\_word) \end{array}$$

(3) $$\begin{array}{l} dispersion\_{difference}^{\sim }speaker\_group*category\\ +\;(category|speaker)+(speaker\_group|target\_word) \end{array}$$

### 2.7 Preanalysis of vowel duration

**Figure A1.**
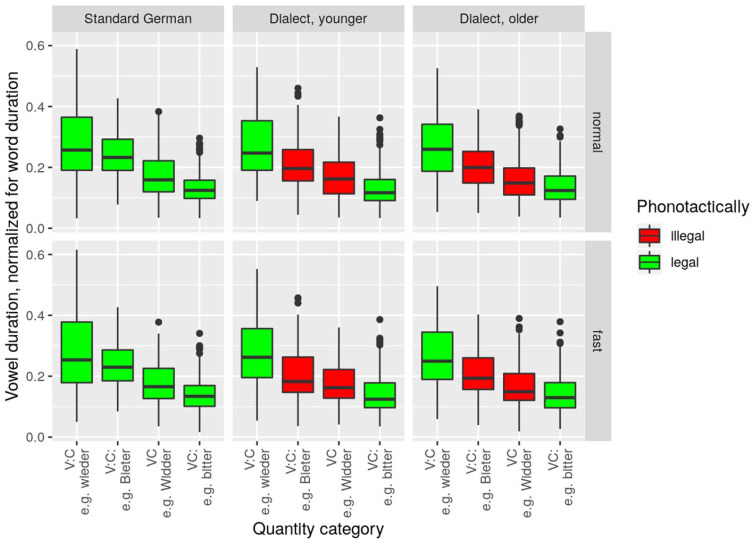
$vowel_{norm}$ (word-normalized vowel duration) per *quantity category, speaker group* (columns) and *speech rate* (rows). Bavarian phonotactically illegal clusters are highlighted in red.

Although the present paper’s focus is on various dependent variables related to consonant duration, an analysis of vowel duration is a prerequisite for this study given that consonant duration in WCB is said to co-vary with the duration of its preceding vowel. A linear mixed-effects model of the structure specified in Equation 1 but with $vowel_{norm}$ instead of $closure_{norm}$ as the dependent variable revealed a statistically significant main effect for *quantity category, F*(3, 21.3) = 5.4, *p* < .01, but not for any other fixed factor and not for any interaction (cf. Figure A1 in the Appendix). This result indicates that (1) vowel duration only varied as a function of the underlying vowel category (i.e., V vs. Vː), but neither as a function of (2) the regional background (i.e., standard vs. Bavarian), nor (3) the age group (older vs. younger). (4) Slight variation in vowel duration as a function of the following underlying consonant category (i.e., C vs. Cː) remained in the realm of intrinsic and predictable phonetic variation without any change in category and—most importantly—the effect size was equally large across speaker groups and conditions. The results of this preanalysis thus not only justify our focus on consonantal measures but also suggest that none of the results regarding the consonantal dependent variables in Section 3 is an artifact of vowel duration.

## 3 Results

### 3.1 Closure duration

**Figure 2.**
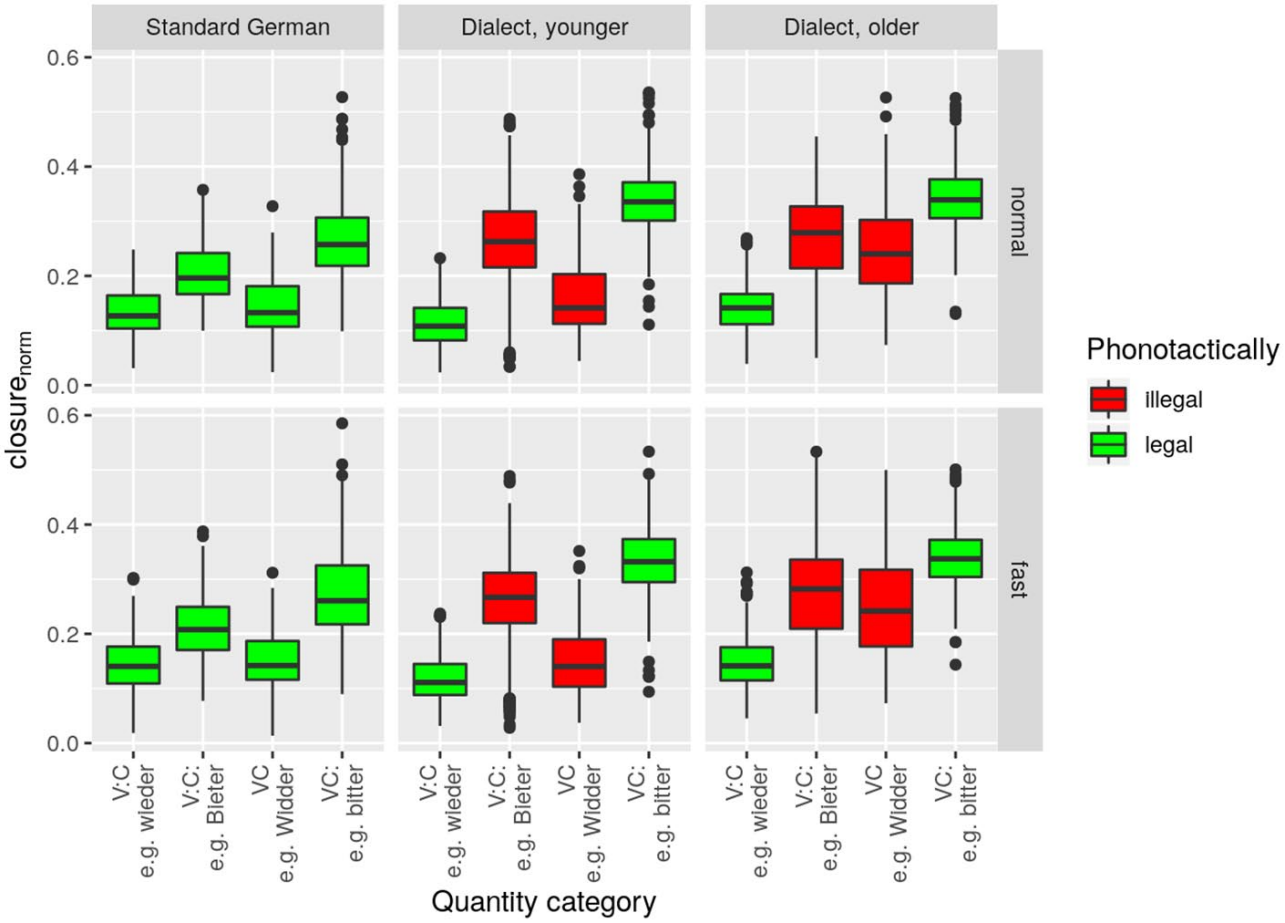
$closure_{norm}$ (word-normalized stop closure duration) per *quantity category, speaker group* (columns) and *speech rate* (rows). Bavarian phonotactically illegal clusters are highlighted in red.

The $closure_{norm}$ data in Figure 2 support the assumption that our control group, the standard speakers, would produce higher closure duration in fortis (Cː) than in lenis (C) stops; and higher closure duration after short vowel (V) than after long vowel (Vː). The younger but not the older dialect speakers exhibit the same pattern as the control group. The key difference is that the older speakers produce short vowel + lenis (VC) words with almost fortis-like closure duration. Figure 2 further suggests that the closure duration in fortis stops is higher for dialect speakers (both younger and older) than for standard speakers.

The linear mixed-effects model revealed statistically highly significant main effects for *speaker group, F*(2, 42.2) = 12.3, *p* < .001, and *quantity category, F*(3, 23.0) = 22.0, *p* < .001, and a significant main effect for *speech rate, F*(1, 36.9) = 4.6, *p* < .05. Statistically significant interactions were revealed between the factors *speaker group* and *quantity category, F*(6, 32.3) = 4.5, *p* < .01, and between *speaker group* and *speech rate, F*(2, 110.5) = 6.6, *p* < .01.

Pairwise comparisons in the model corroborate the observation that the VC category is similar in the control group and the younger dialect speakers, but different in the older dialect speakers (cf. Table 2). As for the fortis categories (VCː and VːCː), the model revealed that $closure_{norm}$ is higher in dialect than in standard, and slightly higher in older dialect than in younger dialect speakers (cf. Table 3; this is also reflected in the statistical significances of pairwise comparisons, with the exception that, for VːCː, the difference between control group and younger dialect speakers does not reach statistical significance). For the VːC category, the difference between the two dialect groups is statistically significant but not the difference between control group and either of the dialect groups.

**Table 2.** Pairwise comparisons of estimated marginal means for $closure_{norm}$ of VC tokens (short vowel plus lenis), averaged across the levels of *speech rate*.

Contrast	Estimate	*SE*	*df*	t.ratio	*p* value
Standard German—Dialect, younger	−0.008	0.023	36.1	−0.352	.9342
Standard German—Dialect, older	−0.100	0.026	33.5	−3.932	.0011
Dialect, younger—Dialect, older	−0.092	0.017	45.3	−5.352	< .0001

**Table 3.** Estimated marginal means for $closure_{norm}$ in fortis consonants (after long vowel [VːCː] and after short vowel [VCː]), averaged across the levels of *speech rate*. Dialect speakers have higher closure duration than standard speakers.

speaker_group	emmean (%)	*SE*	*df*	Lower confidence limit	Upper confidence limit
VːCː					
Standard German	21.0	0.021	31.1	0.167	0.252
Dialect, younger	25.5	0.022	30.3	0.211	0.300
Dialect, older	26.5	0.023	29.4	0.219	0.311
VCː					
Standard German	27.1	0.019	35.2	0.233	0.309
Dialect, younger	33.5	0.020	34.2	0.295	0.375
Dialect, older	34.2	0.020	33.0	0.301	0.383

The speech rate effect found in the data is very subtle, with a fast–normal difference of no more than 0.8 percentage points in any of the three speaker groups. Pairwise comparisons revealed that the estimated difference is 21.4% versus 21.6% for younger and 24.9% versus 25.1% in older dialect speakers (neither of them statistically significant). The estimated difference for the control group is 18.8% versus 19.6% and turned out to be statistically significant (*p* < .001).

These findings indicate that the VC category, which has been described as phonotactically illegal in CB, does indeed not occur in older dialect speakers, but very much so in younger dialect speakers. This can be interpreted as a sound change in progress, by which long stops (Cː) are shortened after short vowels (V), lifting the phonotactical restriction of *no lenis after short vowel*. The findings further suggest that VːCː, contrary to predictions, already exists in the phonological system of all dialect speakers. In line with the literature, the findings indicate that the dialect features higher closure duration in fortis stops than the standard.

### 3.2 Voice onset time

The model with $VOT_{norm}$ as the dependent variable revealed a significant main effect for *quantity category, F*(3, 26.5) = 10.7, *p* < .001, but neither for *speech rate* nor *speaker group*. It also revealed one significant interaction and that is between *quantity category* and *speaker group, F*(6, 34.6) = 7.0, *p* < .001. This suggests that the different speaker groups employ VOT as a cue for phonological quantity in different manners.

**Figure 3.**
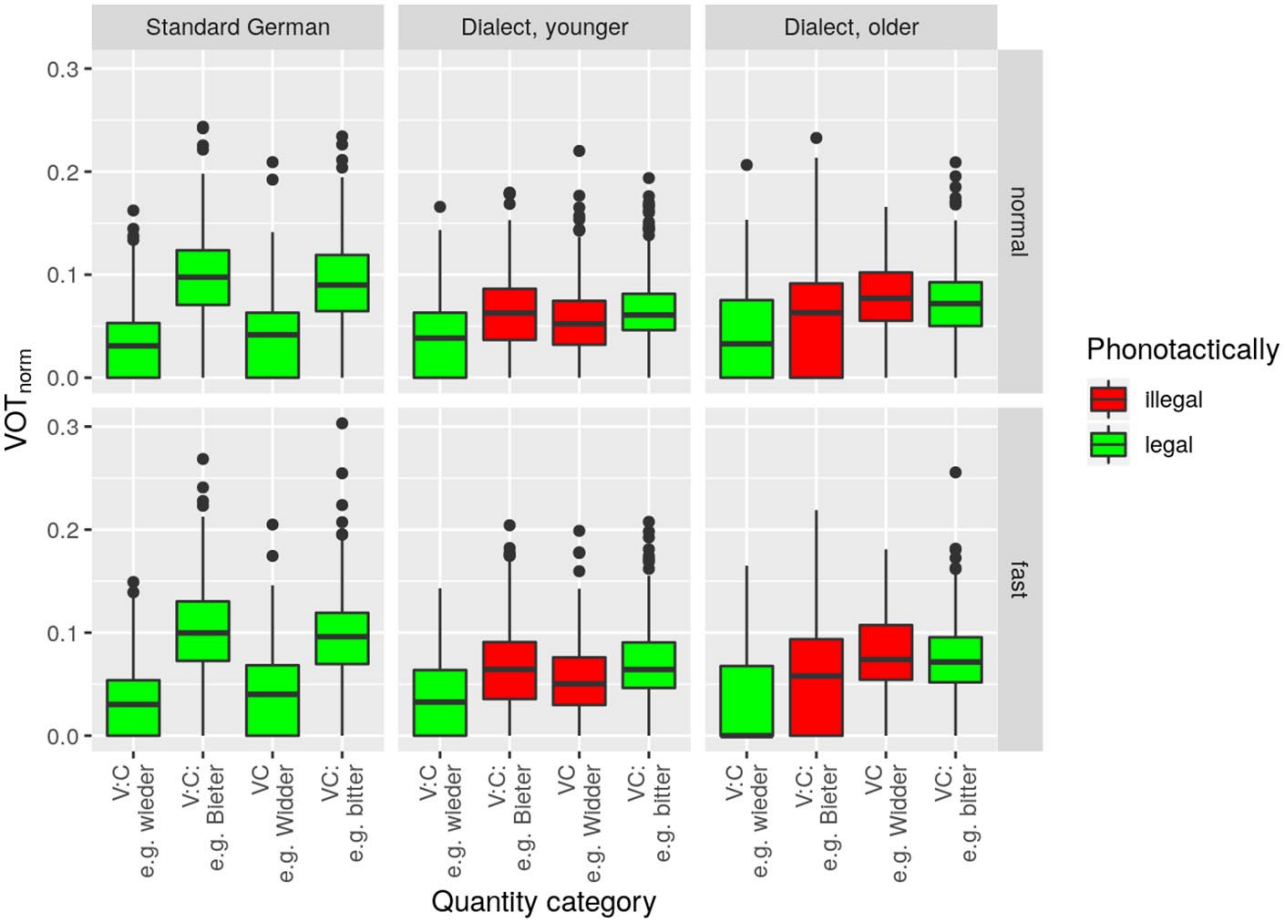
$VOT_{norm}$ (word-normalized voice onset time) per *quantity category, speaker group* (columns), and *speech rate* (rows). Bavarian phonotactically illegal clusters are highlighted in red.

Commensurate with Figure 3 and Table 4, pairwise comparisons show a clear and unsurprising pattern in the standard control group: (1) Fortis consonants have a higher VOT than lenis consonants. (2) The VOT difference turned out significant both after long and short vowels. (3) VOT did not change as a function of speech rate (which is also true of the two dialect groups). This indicates that VOT is a very stable cue for the fortis–lenis contrast in the standard. The younger dialect group exhibits the same general pattern as the standard group, with fortis VOT above lenis VOT in all contexts. However, the fortis–lenis difference is extremely small and only reaches statistical significance after long vowels. This finding suggests that VOT may not yet be used as a robust cue in the production of the phonological fortis–lenis contrast by younger dialect speakers. This interpretation is also supported by the trend that younger dialect speakers produce lower VOT for fortis stops than standard speakers ( $VOT_{norm}$ of 6.4% vs 9.7% after long vowels, *p* < .05; $VOT_{norm}$ of 6.7% vs. 9.3% after short vowels, *p* < .05). Older dialect speakers did not produce a statistically significant VOT difference between fortis and lenis stops, neither after long nor after short vowels, supporting previous accounts by which VOT is said to play no role in Bavarian. Figure 3, however, also shows a greater tendency toward longer VOT after short vowels. This observation reflects the dialectal pattern of stop fortition after short vowels.

**Table 4.** Pairwise comparisons of estimated marginal means for $VOT_{norm}$ , averaged across the levels of *speech rate*.

Contrast	Estimate	*SE*	*df*	t.ratio	*p* value
Standard German:					
VːC–VːCː	−0.065	0.010	40.0	−6.553	< .0001
VC–VCː	−0.050	0.010	34.4	−4.975	.0001
Dialect, younger:					
VːC–VːCː	−0.028	0.009	41.6	−2.959	.0251
VC–VCː	−0.012	0.010	36.0	−1.303	.5671
Dialect, older:					
VːC–VːCː	−0.023	0.013	32.2	−1.818	.2835
VC–VCː	0.006	0.013	28.1	0.434	.9721

Taken together, the findings suggest that VOT was not used as a cue in the older state of the WCB dialect as represented here by the older generation (which is in line with previous accounts, cf. Bannert, 1976; Seiler, 2005; Wiesinger, 1990), but that younger dialect speakers are starting to adopt the cue (which is in line with Kleber’s (2018) observations on these speakers’ regionally accented standard register).

### 3.3 Fast-speech-induced hypoarticulation and variation in closure duration

After having established in Section 3.1 that the VC category is becoming legal in younger dialect speakers we will now consider in more detail whether this sound change in progress is at least to some extent system-internally driven. Given that words such as *Widder* were previously produced with a fortis stop and now exhibit a lenis stop, this is a case of lenition and therefore a particularly relevant candidate for a sound change triggered by fast-speech-induced hypoarticulation. This section will shed light on the effect of speech rate on the fortis–lenis contrast from three different angles: First, we will explore contrast endangerment to show that speech rate variation is indeed an important factor in maintaining the fortis–lenis contrast; second, we will test whether the speech rate effect is disproportionately strong in the category that has changed between generations (VC); and third, we will test whether dispersion is affected disproportionately strongly.

#### 3.3.1 Contrast endangerment

**Figure 4.**
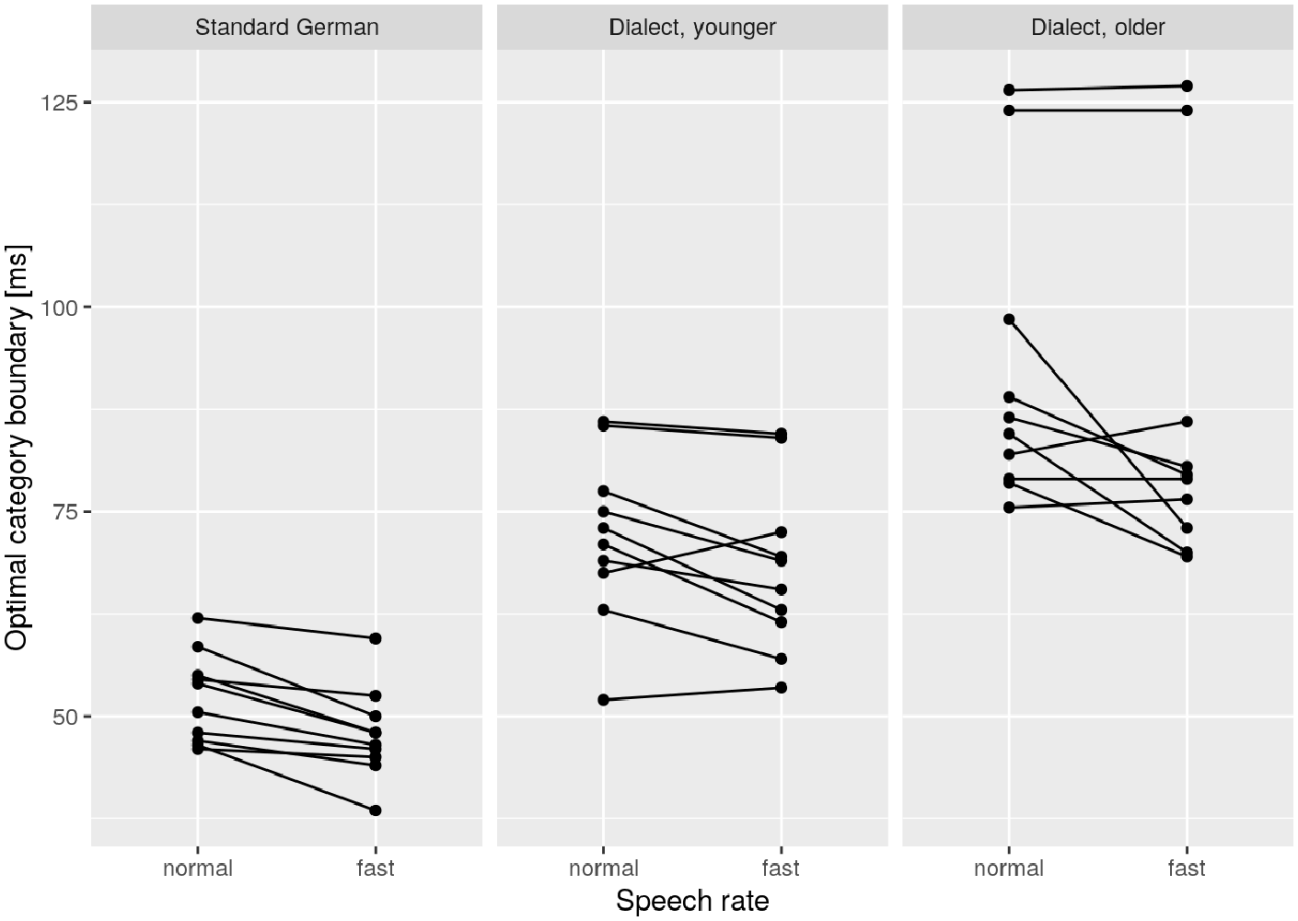
Optimal category boundary between fortis and lenis stop closures per speaker and speech rate. The lines connect the pairs of data points that belong to the same speaker.

Figure 4 shows each speaker’s optimal category boundary between the closure duration of lenis and fortis stops and separately for the two speech rate conditions and speaker groups. It demonstrates a large range of between-speaker variability, especially in the older dialect group, where speakers range from 70 to 127 ms. The younger dialect group ranged from 52 to 86 ms, the control group from 39 to 62 ms. Also commensurate with Figure 4, this between-speaker effect is much larger in our data than the within-speaker effect explored in detail in the previous sections in the form of the fixed effect *speech rate*.

The minor acceleration effect within speakers reported on in Sections 3.1 and 3.2 thus may suggest at first glance that the fortis–lenis distinction is not at risk. However, when taking into account the range of idiosyncratic speech rates observed across speakers, the fortis–lenis contrast perhaps may become endangered after all at the group level, namely then when—following Ohala (1993b) and Harrington et al. (2008)—speakers-turned-listeners do not compensate for speech-rate-induced variation in closure durations unknown to them; that is, values outside their own scope of rate-induced variation.

#### 3.3.2 Lenition in fast speech

The older dialect speakers have a long consonant in the VC, VCː, and VːCː words. The younger speakers have retained the long consonants in VCː and VːCː, but have shortened those in VC. If this consonant shortening had been caused by failing compensation for speech rate, we would expect the long consonant in the older speakers’ VC to be less stable across speech rates (and therefore, we suppose, harder to normalize) than in the other two categories. This should surface in a stronger effect of *speech rate* on (the already word-normalized measure) $closure_{norm}$ in VC, compared with the other two categories. We hypothesize:

In the fast speech condition, older dialect speakers reduce $closure_{norm}$ in VC but not in VːCː and not in VCː—possibly to the extent that VC is merged with VːC (with VːC being the only category where older speakers have a short consonant to begin with).

However, pairwise comparisons in our model from Section 3.1 revealed a statistically significant speech rate effect only for 3 out of 12 pairs (3 speaker groups × 4 quantity categories), and even these effects are tiny: VːCː in older dialect speakers (26.1% in normal vs. 26.9% in fast, *p* < .05), VːC in younger standard speakers (13.5% vs. 14.4%, *p* < .01), and VCː in younger standard speakers (26.6% vs. 27.6%, *p* < .01).

These findings suggest that faster speech rate only shortens stop closures in a manner exactly proportional to word shortening. Contrary to our prediction, older dialect speakers did not shorten closure phases (i.e., lenite) in the VC category disproportionately strongly.

#### 3.3.3 Dispersion difference

To extend the test whether older dialect speakers show signs of instability in fast speech, we measured the *dispersion difference* (cf. Section 2.5.3) based on $closure_{norm}$ in the data. We expected unstable phonological conditions to be associated with categories that are more dispersed in fast speech than in normal speech.

**Figure 5.**
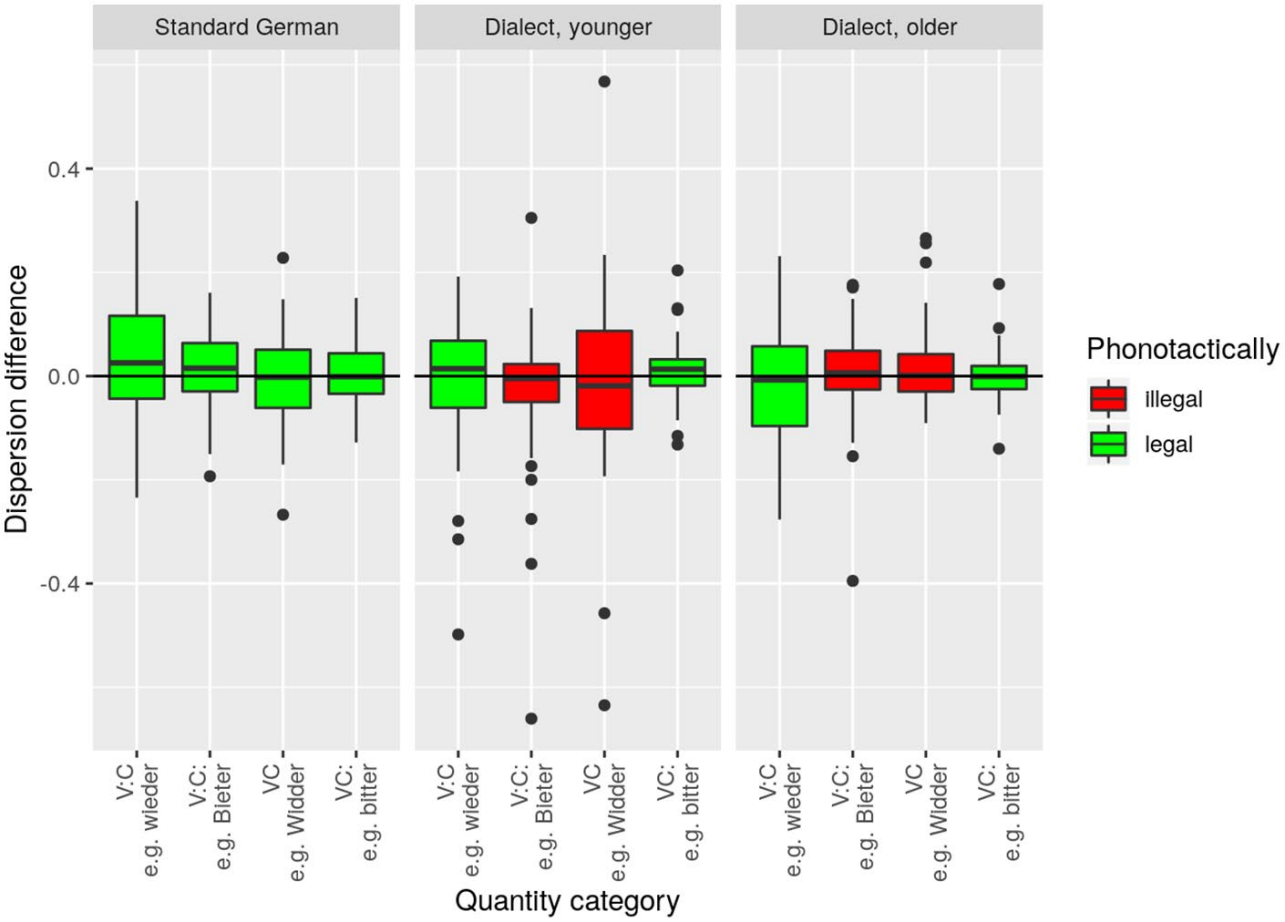
*Dispersion difference*. Each data point represents the dispersion difference per speaker and word. Positive values indicate more dispersion of the acoustic parameter $closure_{norm}$ in the fast condition as opposed to the normal-paced condition. Bavarian phonotactically illegal clusters are highlighted in red.

However, commensurate with Figure 5, no significant group differences were found in how speech rate affects the dispersion of words at the level of phonological categories. The corresponding statistical model (with *speaker group* and *phonological category* as fixed factors) did not reveal a significant main effect or interaction. Together with the result regarding lenition in fast speech (Section 3.3.2), the outcome of the statistical analysis of *dispersion difference* thus suggests once more that there is no compelling evidence of instability in the dialect speakers’ fast speech. Figure 5 nevertheless shows considerably more outliers—particularly (1) in younger WCB speakers and (2) interestingly in the range of negative values. While (2) points to slightly more variation in normal speech as opposed to fast speech, (1) indicates a greater tendency toward variation in the dialect group as opposed to the group of standard speakers. This latter result (1) is thus in line with our finding of greater between-speaker variability in the optimal category boundary.

### 3.4 Word-specific differences in category overlap

**Figure 6.**
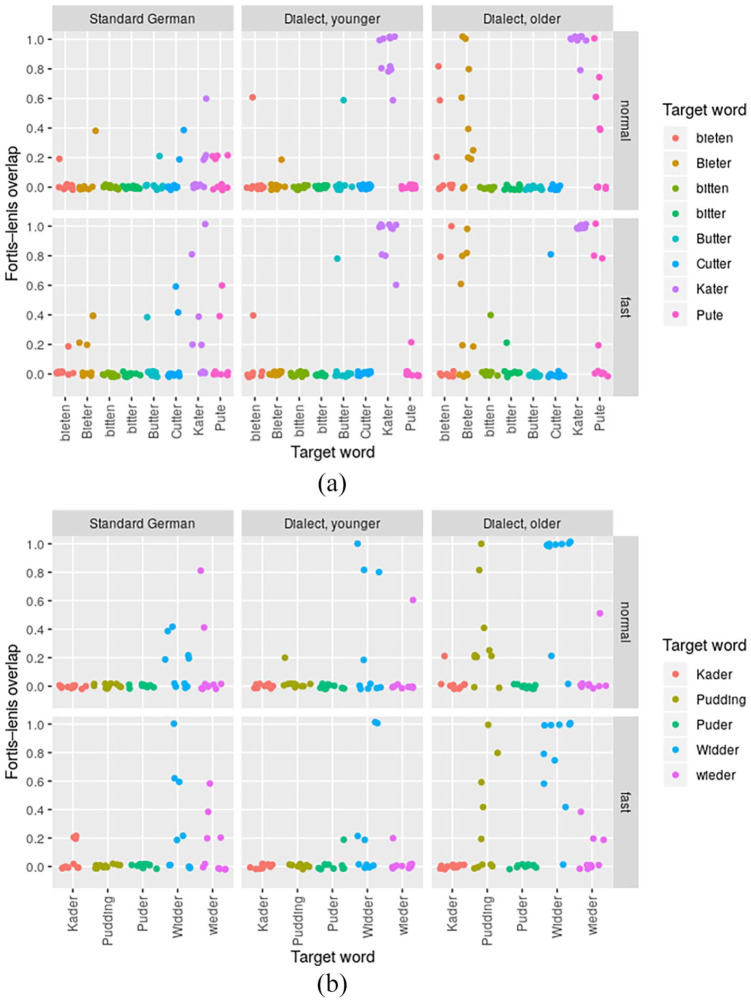
Fortis*–*lenis overlap per speaker, word, and speech rate for alveolar words. Part (a) includes data from fortis words (e.g., *Kater*) that were compared with a baseline set of lenis words. Part (b) includes data from lenis words (e.g., *Pudding*) that were compared with a baseline set of fortis words. The coordinates may slightly deviate from their real values to better separate visually data points that are close together; this was done using ggplot’s geom_jitter function.

**Figure A2.**
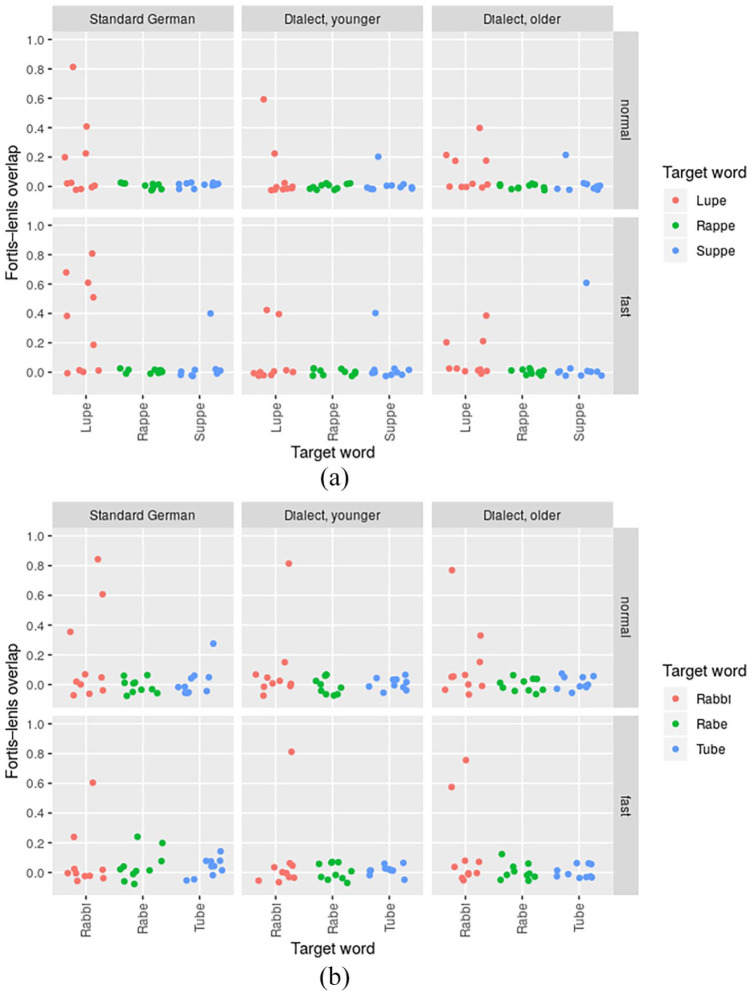
Fortis*–*lenis overlap per speaker, word, and speech rate for labial words. Part (a) includes data from fortis words (e.g., *Lupe*) that were compared with a baseline set of lenis words. Part (b) includes data from lenis words (e.g., *Tube*) that were compared with a baseline set of fortis words. The coordinates may slightly deviate from their real values to better separate visually data points that are close together; this was done using ggplot’s geom_jitter function.

**Figure A3.**
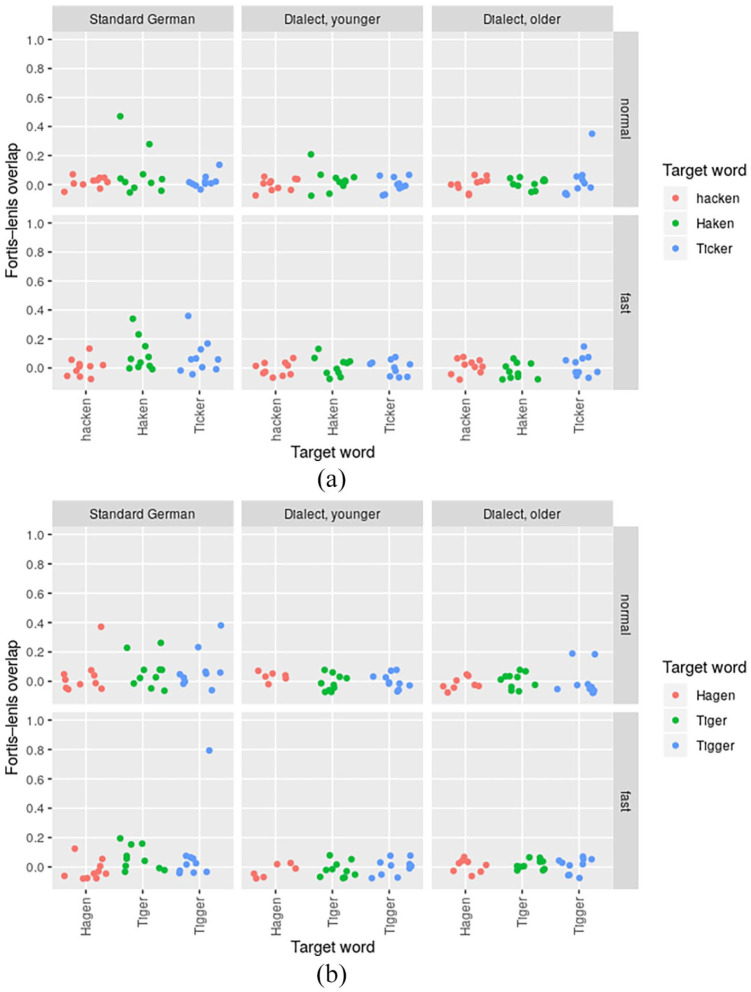
Fortis*–*lenis overlap per speaker, word, and speech rate for velar words. Part (a) includes data from fortis words (e.g., *Haken*) that were compared with a baseline set of lenis words. Part (b) includes data from lenis words (e.g., *Tiger*) that were compared with a baseline set of fortis words. The coordinates may slightly deviate from their real values to better separate visually data points that are close together; this was done using ggplot’s geom_jitter function.

In this analysis, which is last in the paper but preliminary in nature, we will explore our data on a word-by-word basis. In Figures 6, A2 and A3 (the latter two being in the Appendix), we use the *fortis–lenis overlap* (see Section 2.5.4) to show how often words were realized with a closure duration typical of the stop category they do not include. The fortis–lenis overlap can take values between 0 and 1; in the present analysis, it can specifically take the values 0, 0.2, 0.4 and so on, as we have five repetitions of each word per speaker and condition.^14^ 0 in a fortis word means that no tokens were produced lenis-like, whereas 1 means that all tokens were produced lenis-like. In a lenis word, 0 means that no tokens were produced fortis-like, whereas 1 means that all tokens were produced fortis-like. “Fortis word” and “lenis word” refer to words that contain the respective kind of stop phoneme in Standard German. We consider a fortis–lenis overlap between 0 and 1 to be a sign of unstable categories. The speech materials of the present analysis in combination with the vowel pre-analysis in Section 2.7 allow the additional interpretation that for the vowel-plus-stop sequences illegal in WCB, values closer to 1 mean the tokens were realized conservatively and values closer to 0 mean the tokens were realized innovatively by the respective dialect speaker.

An initial analysis of all 25 words in our corpus revealed that speaker-group differences in the word-specific *fortis–lenis overlap* only emerged in words with alveolar stops (henceforth alveolar tokens), but not in words with labial or velar stops (henceforth labial and velar tokens, respectively). More precisely, while *fortis–lenis overlap* values for non-alveolar tokens were almost always around 0, with just a few outliers (see Figures A2 and A3 in the Appendix), suggesting no or very little overlap, values for alveolar tokens showed much more variation and reached the value of 1 for some words. Although this result may look at first sight like an effect of place of articulation, it may very likely instead be linked to the fact that the number of alveolar tokens was twice the number of velar and labial tokens, respectively (see Section 2.2). That is, alveolar tokens may have provided a better sample size for detecting word-specific differences in the fortis*–*lenis-overlap. In the remainder of this section, we will thus concentrate on a description of these word-specific differences in alveolar tokens; in Section 4, we will resume considering the possibility of place of articulation being a potential phonological factor in the diachronic process of this sound change.

Figure 6 depicts the fortis–lenis overlap for each individual word with an alveolar fortis stop in part (a) and an alveolar lenis stop in part (b). We can see that the word *Kater* “tomcat” is almost exclusively produced as lenis by the older dialect speakers (fortis–lenis overlap being 1 for 9 out of 10 speakers at normal speech rate), but has a slight tendency toward fortis in some younger dialect speakers (with one speaker exhibiting a fortis–lenis overlap of 0.6, four speakers a fortis–lenis overlap of 0.8 and five speakers a fortis–lenis overlap of 1 at normal speech rate; that is, 3 of 5, 4 of 5, and 5 of 5, respectively, of the speaker’s repetitions exhibiting acoustic values typical of lenis stops). For the words *bieten, Bieter, Pute* (“to bid,” “bidder,” “turkey hen”), the older dialect group is very heterogeneous in whether they have lenis or fortis stops. The younger dialect speakers, however, have pretty much settled on fortis.

Among the lenis words, *Pudding* “Pudding” behaves similarly to *bieten, Bieter, Pute*: While the older dialect speakers are still very heterogeneous in whether they produce fortis or lenis stops, the younger dialect speakers have settled on lenis. For *Widder* “ram,” the older dialect speakers produce fortis rather consistently at normal speech rate (with 8 out of 10 speakers exhibiting a fortis–lenis overlap of 1), but heterogeneously both fortis and lenis at fast speech rate. The younger dialect speakers are inconsistent for *Widder*. Interestingly, the Standard German speakers are also inconsistent for this word, even though we expected no inconsistencies at all for Standard German.

The two *speech rates* mostly exhibit very similar values of fortis–lenis overlap. One notable exception is the word *Widder* “ram,” where older speakers are considerably less consistent in fast than in normal-paced speech.

These findings suggest that the change is governed by lexical diffusion, at least in words with alveolar stops. That is, in CB both /d/ and /t/ surface in more conservative quantity patterns in some words (e.g., as long [t] after short [i] in *Widder* and as short [d] after long [aː] in *Kater*) but in more innovative quantity patterns in others (e.g., as short [d] after short [ʊ] in *Pudding* and as long [t] after long [uː] in *Pute*). The phonetic implementation even varies within speakers as shown by *fortis–lenis overlap* values far away from both 0 and 1 for certain words and speakers, suggesting within-speaker variation rather than only between-speaker variation in this process.

## 4 Discussion

Our three main aims in this paper revolved around an ongoing change in a German dialect. We aimed (1) to test whether long vowel plus fortis stop sequences emerge in the WCB dialect (as shown for ECB in Moosmüller and Brandstätter (2014) and suggested by WCB speakers’ usage of the regionally accented standard register (Kleber, 2018, 2020)), (2) to test whether this change extends to short vowel plus lenis stop sequences. Finally, we aimed (3) to model naturally occurring fast speech to test whether such a change can also be related to language-internal factors (in this case, fast-speech-induced hypoarticulation). To achieve these aims, we used a newly collected corpus of dialect recordings that included all four phonotactic categories involved in the sound change (including the previously understudied short vowel plus lenis stop [VC] combination). In the corpus, we further required speakers to doubtlessly be operating in a dialect register in spite of the laboratory setting. We achieved this by writing down the prompts in non-orthographic forms and having speakers recite those from memory shortly after seeing them in writing.

The five main findings are as follows: Stop closure duration indicates that (1) the combination of short vowel plus lenis consonant has become legal in younger dialect speakers, and (2) the combination of long vowel plus fortis is legal even in older dialect speakers. As to VOT, we found that (3) younger dialect speakers’ usage of this acoustic parameter in speech production is between that of older dialect speakers and younger standard speakers. (4) Analyses of two acoustic parameters and three further derived measures did not yield a plausible reason why fast-speech-induced hypoarticulation can be considered a trigger of the observed sound change (but see below). Furthermore, (5) analyses of such derived measures, specifically the fortis–lenis overlap, showed that some words exhibit markedly more between-speaker and within-speaker variation than others.

Our results confirm that the observations made for the ECB dialect (see Klingler et al., 2017; Moosmüller & Brandstätter, 2014) and the WCB regional accent (Kleber, 2018, 2020) (i.e., Standard German, but noticeably produced by WCB dialect speakers), also hold true for the WCB dialect: The combination long vowel plus fortis consonant (VːCː) already forms part of the older dialect speakers’ phonological system (participants of the present study born 1944–1968). Our results further confirm that the other supposedly illegal combination, short vowel plus lenis consonant (VC), does indeed not occur in the older speakers, but it does occur in the younger speakers (participants of the present study born 1987–1997). On the whole, this is in line with the results reported in recent years (Kleber, 2018, 2020; Moosmüller & Brandstätter, 2014; Schikowski, 2009) suggesting that the complementary length feature of CB dialects is converging toward the standard: Both lenis and fortis consonants have existed before, but the combinations of short vowel plus lenis consonant (VC) and long vowel plus fortis consonant (VːCː) are new options in the dialect’s phonology.

This finding of three and four phonotactic combinations in older and younger WCB speakers, respectively, further suggests that the VːCː sequence must have emerged in the phonological system of CB prior to that of the VC sequence. As the VC category was not empirically studied before, this finding can, unfortunately, only be assessed by means of between-study comparisons for the VːCː category. The non-existent speaker-group difference for this category is, on one hand, in line with the data presented in Moosmüller and Brandstätter (2014) where the VːCː category, too, was already present in the system of ECB. On the other hand, the result appears at first sight to contradict the findings described in Kleber (2018, 2020), where two age cohorts of WCB dialect speakers very similar to ours differed significantly in the extent of co-variation between vowel and stop duration in VːCː combinations when they are operating in a regionally accented standard register. However, the results from the present and the preceding studies may not be conflicting after all as both previous studies showed that older WCB speakers did produce VːCː combinations with duration patterns that are markedly different from VCː and VːC sequences. The older and younger WCB speakers investigated in the two previous studies only differed in the fine phonetic implementation of the phonological sequence such that dialectal traces were present to a significantly greater extent in the older speakers’ productions compared with those of the younger speakers. Two reasons may account for the deviating age-group effects. First, the large categorical age-group difference for VC combinations in the present study that investigated for the first time all four combinations may have masked potential gradient age-group differences for VːCː combinations similar to those found in Kleber (2018, 2020) that investigated the vowel length and the fortis*–*lenis contrast separately. Second, the words investigated in the previous studies differed largely from those analyzed in the present study. Given our findings regarding word-specific differences in the realization of VːCː combinations (see below for a discussion of lexical diffusion), such a between-study difference in the design may also be responsible for differences regarding the phonetic implementation of categories.

In addition to the changes regarding the co-variation between vowel and stop duration, our results also indicate that usage of VOT as a cue to the lenis–fortis contrast in stops is becoming stronger in WCB. While the younger dialect speakers still produce shorter VOT in fortis stops and also a smaller degree of separation between lenis and fortis (in terms of VOT) than the standard speakers, they also deviate clearly from the older dialect speakers, who produce yet shorter VOT and almost no separation between lenis and fortis (again, in terms of VOT). This is again in line with Kleber (2018), who found that younger speakers of the WCB regional accent (rather than dialect) employ VOT in their production (and indeed also perception) more strongly than older speakers. Taken together, then, these apparent-time observations also point toward the emergence of VOT in WCB as an acoustic cue to the fortis–lenis contrast that is not only used in the regional accent but also in the dialect. It is very likely that this diachronic development, too, is linked to language contact and borrowing given that between-speech-rate differences were not significant—and very consistently so across speaker groups (see below for further discussion). This change may additionally be linked to the emergence of the fortis–lenis contrast in syllable-initial position which has been reported for the regional accent of WCB (Kleber, 2018) and ECB dialects (Luef, 2020), though no conclusion as to their potential mutual influence can be drawn at this point.

Moreover, the apparent asynchronous change of the two acoustic cues—with duration but not yet VOT patterns in younger dialect speakers equaling those found in the standard—suggests that the trajectory of this sound change is, like many others, gradual (e.g., Ruch & Harrington, 2014 on metathesis, Kirby, 2014 on tonogenesis) and involves various transition phases in which different acoustic cues to a phoneme or phonemic contrast loose or gain weight (see, for example, Beddor, 2009; Harrington et al., 2012; Hyman, 1976; Ohala, 1993a). Although a number of studies have discussed the potential time course of intrinsically motivated gradual sound changes (e.g., Beddor [2009] on vowel nasalization, Kirby [2014] on tonogenesis), externally motivated sound changes as a result of dialect leveling have long been treated as being inherently categorical and abrupt (Labov, 1994). The finding of the present study, however, adds to the increasing evidence for gradual sound changes as a result of dialect leveling (e.g., Bukmaier et al., 2014; Kerswill, 2002).

Our research was focused on the phoneme level, grouping all words from the corpus into the four categories VːC, VːCː, VC, and VCː. A close look inside these groups reveals that, on the whole, they are pronounced homogeneously within each speaker group. However, one word in particular—*Kater* “tomcat”—contradicts this generalization. Like, for example, *Haken* “hook” and *Lupe* “magnifying glass,” *Kater* was in the VːCː group due to its canonical pronunciation in Standard German. However, in both the older and the younger dialect speakers, the closure duration in this word severely stands out from the other words in the VːCː group. The observed lenis-like realizations of the alveolar stop in *Kater* are in line with the grammar of WCB and exactly what many dialect researchers would expect. However, the other words in the group did not match this expectation, often clearly exhibiting both a phonetically long vowel and long stop. There were other deviations from homogeneity as well. The words *bieten* “to bid,” *Bieter* “bidder,” and *Pute* “turkey hen” exhibited considerable within-speaker and between-speaker variation in the older dialect group, with some speakers leaning toward a fortis stop, some toward a lenis stop, and some without a clear preference for either. This observation further suggests that the spread of this gradual sound change in progress (see above) is determined to some extent by the mechanism of lexical diffusion. While this mechanism traditionally has been linked to abrupt sound changes in the context of dialect borrowing (Labov, 1994), our findings lend further support for the claim put forward in Kerswill (2002) that externally driven, gradual sound changes may also spread irregularly; that is, via lexical diffusion (see also Bang et al., 2018, for a mixture of regularity and irregularity in an internally driven sound change). Future studies therefore must consider lexical diffusion as a mechanism for the observed sound change, but they should then test a large set of words, apt for investigating the spread of the change through the lexicon as well as potential effects of lexical frequency on lexical diffusion (cf. Bang et al., 2018; Todd et al., 2019).

Although our descriptive findings suggest that some form of lexical diffusion must be at play, the present study does not allow for further generalization regarding word frequency because the limitations of the speech materials (for reasons discussed above in Sections 2.2 and 3.4) did not allow to include it as a predictor variable. Perhaps with the exception of four words (*Kater, Rappe, Tigger, wieder*; i.e., one item per category) the log frequency per million was comparable across most words. However, we want to point out an interesting observation that is in line with previous studies on word frequency and lexical diffusion. It appears promising to follow this path of investigation further: The most conservative quantity pattern within the formerly illegal VːCː combination was observed for *Kater*, by far the word with the highest log frequency per million value (see also Note 4). According to the Frequency Actuation Hypothesis a sound change occurs last (Phillips, 1984) and progresses more slowly (Todd et al., 2019) in high-frequency words only if it is not triggered physiologically. Otherwise—that is, in physiologically motivated sound changes, as is the case with diachronic vowel nasalization (Beddor, 2009; Carignan et al., 2021) or high back vowel fronting after coronal consonants (Harrington, 2007; Harrington et al., 2011; Ohala, 1981)—high-frequency words are affected first and progress faster than low-frequency words. This line of argument is consistent with our conclusion that the present change is primarily motivated by external as opposed to internal factors. Moreover, by attributing an important role to the listener, Todd et al. (2019) in their listener-based sound change model within an exemplar-framework (cf. Pierrehumbert, 2016) demonstrated that low-frequency words change faster in phonemic splits compared with other changes such as mergers; that is, in the same type of change as the one under investigation of the present study. However, any observation based on the present materials should be treated as a very preliminary and tentative indication of a faster change of lower-frequency words in this change via lexical diffusion. Having established—based on fine-phonetic differences between minimal pairs—that the dialectal feature of complementary length is being diachronically reversed in WCB, a future study investigating the effects of word frequency in this change should specifically investigate non-minimal pairs given that minimal pairs alone are very unlikely to drive this change nor do they provide sufficient material to study frequency effects (cf. Todd et al., 2019, p. 16).

Given that only alveolar tokens show traces of lexical diffusion one could infer that the change is also influenced by the phonological factor place of articulation—as pointed out by one of the reviewers. Accordingly, the place features labial and velar increased a word’s likelihood to be affected by an innovation, whereas the place feature alveolar would delay an innovation in the form of a prolonged use of conservative pronunciations. Whether or not the change in labials and velars has progressed via lexical diffusion can no longer be stated for the words analyzed in the present study. Any discussion of the role of place of articulation in this postvocalic consonantal quantity change, however, will ultimately include the issue of the frequency in phonotactic co-occurrence of long and short vowels before fortis and lenis stops (cf. Féry, 2003; Kleber et al., 2010) which has led to the imbalance of alveolar versus non-alveolar tokens in the present corpus in the first place. The higher frequency of long vowels before fortis alveolar stops compared with the lower frequency of long vowels before labial or velar stops may lead to an unexpected advanced change in the rarer sequence. Whether or not lexical diffusion may also be mediated by phonological features remains an open question at this point and should be investigated in future studies on the basis of different speech materials perhaps from other languages (to avoid the presupposition of the lexicon as found in German).

Kleber (2020) discussed in more detail the plausibility of the change investigated in this study (and other changes for that matter, for example, Harrington et al. (2012), Bukmaier et al. (2014)) to be driven by external factors (dialect convergence toward a standard) rather than internal factors. With the present paradigm of modeling naturally occurring fast speech, we put to the test an alternative explanation based on Kohler (1984) and Ohala (1993a): fast-speech-induced hypoarticulation providing a phonetic bias to diachronically lenite fortis phonemes. Such an investigation was warranted given that changes toward a standard variety are all too often attributed post hoc to externally motivated convergence, and also given the evidence of such a change to be internally rather than externally motivated presented in Rathcke and Stuart-Smith (2016). One of our main findings is that the four words that make up the VC group (e.g., *Pudding*) have a word-medial fortis stop in the older dialect speakers but that stop is lenis in the younger dialect speakers. If this shortening had been triggered by the above-mentioned phonetic bias, we would have expected to find synchronic lenition in the older group’s fast VC words (more than in the other word groups, where no diachronic lenition has been observed in the past decades), particularly in the consonants. However, we did not find this kind of lenition. We, therefore, extended our analyses to see if any instabilities regarding the *optimal category boundary* or in form of a greater *dispersion difference* between fast and normal speech could be found in the words affected by the change, either in the older dialect speakers (to suggest a phonetic bias leading to the change) or in the younger speakers (as a sign that the new phonology has not yet stabilized). With the exceptions of an enhanced between-speaker variability regarding the optimal category boundary, particularly in the older WCB group, more outliers in the *dispersion difference* in particular in the younger WCB group (though in the opposite direction), and more variation in *fortis–lenis overlap* of Widder tokens in older WCB speakers, the results for the dependent variables specifically derived for testing speech rate effects did not suggest that fast-speech induced hypoarticulation had a considerable effect on the stability of the implementation of VC and VːCː combinations, respectively. Following Mitterer (2018) we may then conclude that speech rate variation is not large enough in WCB to endanger the phonemic fortis*–*lenis contrasts. However, the findings for between-speaker variation regarding the optimal category boundary and the dispersion difference as well as the speaker- and word-specific finding for the *fortis–lenis overlap* are indeed important for a future testing of whether or not speech rate variation has the potential for the perceptual endangerment of a phonemic category (see Baker et al., 2011, for the importance of the individual in the origin of diachronic change). Following Ohala (1993b) and Harrington et al. (2008), one potential hypothesis would be to test whether some listeners falsely classify stimuli with duration patterns outside their own scope of rate-induced variation.

Regardless of the implications for speech perception, potential greater speech-rate effects in production may have been concealed for two other reasons: (1) Consonants in general have been found to be affected by speech rate increases much less than vowels (Gay, 1981) and (2) short phonemes (in our case the stressed vowels in the first syllable of the VC words) have been found to be affected less than long phonemes (Hoole & Mooshammer, 2002). However, these studies, just like ours, *did* find shortening effects on consonants and on lax vowels. The effects are simply small in size, suggesting that phonemic categories are not necessarily endangered by speech rate (Mitterer, 2018). Moreover, using a similar paradigm as ours, Bukmaier and Harrington (2016), too, found no compelling evidence for fast speech rate to trigger a fricative-merger in some Polish dialects, although dialect convergence toward a standard as an alternative explanation could be ruled out in their case. Our findings for the two speech-rate conditions together with those results suggesting lexical diffusion, therefore, lead us to conclude that the apparent sound change in progress investigated in the present study is more likely to be directly triggered by means of language contact (convergence toward a standard), as hypothesized in Kleber (2020), with no need for a specific phonetic bias in the older state of the language to foster the change. This externally driven change, nevertheless, appears to be gradual and whether or not the misperception of speech rate (Ohala, 1993a) and/or a change in perceptual trading relationships (cf. Beddor, 2009) plays a role in the spread of this change is yet to be tested.

## Supplemental Material

sj-csv-1-las-10.1177_00238309221127641 – Supplemental material for Fast-Speech-Induced Hypoarticulation Does Not Considerably Affect the Diachronic Reversal of Complementary Length in Central Bavarian Supplemental material, sj-csv-1-las-10.1177_00238309221127641 for Fast-Speech-Induced Hypoarticulation Does Not Considerably Affect the Diachronic Reversal of Complementary Length in Central Bavarian by Markus Jochim and Felicitas Kleber in Language and Speech

sj-csv-2-las-10.1177_00238309221127641 – Supplemental material for Fast-Speech-Induced Hypoarticulation Does Not Considerably Affect the Diachronic Reversal of Complementary Length in Central Bavarian Supplemental material, sj-csv-2-las-10.1177_00238309221127641 for Fast-Speech-Induced Hypoarticulation Does Not Considerably Affect the Diachronic Reversal of Complementary Length in Central Bavarian by Markus Jochim and Felicitas Kleber in Language and Speech

## Appendix

### Carrier sentences, Standard German

The following carrier sentences were used with the Standard German participants:

- Ich wollte wieder sagen. “I wanted to say ‘again.’”
- Er will doch Bieter werden. “He wants to become a bidder.”
- Er will mal Widder streicheln. “He wants to stroke rams some time.”
- Das hat doch bitter geschmeckt. “This tasted bitter.”
- Er kann die Lupe brauchen. “He can make use of the magnifying glass.”
- Er will die Tube nehmen. “He wants to take the tube (packaging).”
- Ich will die Suppe kochen. “I want to cook the soup.”
- Er muss auf Hagen warten. “He has to wait for Hagen.”
- Sie muss noch Haken kaufen. “She has yet to buy hooks.”
- Er hat noch hacken müssen. “He had yet to chop.”
- Er hat den Kader besetzt. “He picked players for the squad.”
- Er will den Kater füttern. “He wants to feed the tomcat.”
- Er muss den Cutter kaufen. “He has to buy the cutter.”
- Ich soll doch Rabe lesen. “I ought to read ‘raven.’”
- Er wollte Rabbi werden. “He wanted to become a rabbi.”
- Ich soll doch Rappe lesen. “I ought to read ‘black horse.’”
- Sie muss noch Puder kaufen. “She has yet to buy powder.”
- Er muss die Pute kaufen. “He has to buy the turkey hen.”
- Sie wollte Pudding kochen. “She wanted to cook pudding.”
- Ich muss doch Butter kaufen. “I have to buy butter.”
- Sie will den Tiger füttern. “She wants to feed the tiger.”
- Sie hat dich Tigger genannt. “She called you Tigger.”
- Ich soll den Ticker nehmen. “I ought to take (use) the ticker.”
- Er hat doch bieten müssen. “He had do bid.”
- Sie hat euch bitten müssen. “She had to ask you a favor.”

### Carrier sentences, Western Central Bavarian

The following carrier sentences were used with the dialect participants:

- I woit doch wieder sagn. “I wanted to say ‘again.’”
- Ea wui doch Bieter wern. “He wants to become a bidder.”
- Er wui no Widder streicheln. “He still wants to stroke rams.”
- Des hod doch bitter gschmeckt. “This tasted bitter.”
- Ea konn a Lupen bracha. “He can make use of a magnifying glass.”
- Ea wui de Tuben nehma. “He wants to take the tube (packaging).”
- I wui a Suppen kocha. “I want to cook soup.”
- Ea muass aufn Hagen watn. “He has to wait for Hagen.”
- Sie muass no Haken kaffa. “She has yet to buy hooks.”
- Ea hod no hacken miassn. “He had yet to chop.”
- Ea hod den Kader bsetzt. “He picked players for the squad.”
- Ea wui den Kater fuadan. “He wants to feed the tomcat.”
- Ea muass an Cutter kaffa. “He has to buy a cutter.”
- I soi eich Rabe voasagn. “I ought to say ‘raven’ to you.”
- Ea woit Rabbi wern. “He wanted to become a rabbi.”
- I soi eich Rappe voasagn. “I ought to say ‘black horse’ to you.”
- Sie muass an Puder kaffa. “She has yet to buy powder.”
- Ea muass a Pute kaffa. “He has to buy the turkey hen.”
- Sie woit an Pudding kocha. “She wanted to cook pudding.”
- I muass doch Butter kaffa. “I have to buy butter.”
- Sie wui no Tiger fuadan. “She still wants to feed tigers.”
- Sie hod di Tigger gnennt. “She called you Tigger.”
- I soi den Ticker nehma. “I ought to take (use) the ticker.”
- Ea hod doch bieten miassn. “He had do bid.”
- Sie hod eich bitten miassn. “She had to ask you a favor.”

## Notes

1. Hinderling (1980) notes that *Pudding* is a loan from Standard German.

2. Standard German can be broadly divided into Northern and Southern Standard German. All speakers, except perhaps trained speakers, can be assigned to one of the two groups. Speakers of Southern Standard German exhibit, for example, [s] for word-initial /z/ phonemes but apart from such minor deviations from the dictionary pronunciation, they cannot be identified perceptually as speakers of a regional accent by phonetically naive people (i.e., they cannot be classified more precisely than northern vs. southern). More importantly, there are no noticeable differences between the northern and the southern standard variety regarding the vowel length or the fortis–lenis contrasts.

3. Many Western Central Bavarian (WCB) dialect speakers are able to use a standard register. When they do, however, they can be divided into speakers of a regionally accented standard (i.e., phonetically naive people can classify them more precisely than northern vs. southern) and speakers of Southern Standard German.

4. This test was conducted because the prerequisite of a normal distribution was violated in the group of illegal patterns, caused by the higher-frequency word *Kater*. A Welch two-sample *t*-test with the same fixed factor and dependent variable based on a data set of 24 words, with *Kater* having been excluded from the analysis, revealed again no significant difference between groups, t(19.9)=−0.8,p>0.05 . In this comparison, the mean lgSUBTLEX value for words with a legal pattern in Bavarian was 2.03 and the mean value for words with an illegal pattern 1.7.

5. We could have conducted a picture-naming experiment, but these typically yield lexically and prosodically less uniform participant responses.

6. We had an irregularity in one of the minimal sets (Tube, Lupe, Suppe), which we spelled with an added -n in the dialect speakers’ prompts. This resembles a typical dialectal pronunciation and, indeed, our dialect participants realized a nasally released stop instead of a post-stop schwa in almost all cases. This irregularity went unnoticed when preparing the experiment. We did not encounter any problems arising from that in our analyses of closure or aspiration duration because all measurements were done equally across words and the distribution of nasal releases was similar across V + C-sequence. The above-mentioned words line up with other minimal pair words ending in a nasal (e.g., *bieten* “to bid”) and typically realized, in both varieties, without a schwa.

7. With some participants, we repeated the fast condition, because they failed to accelerate their speech measurably in the first try.

8. Note that the data discussed here are a subset of a larger corpus collected and annotated by three research institutions with several labelers involved.

9. The value of 280 was arbitrarily chosen, yet it represents a plausible number for the upper limit based on the distribution of the data.

10. In the present study, this measure has only been applied to $closure_{norm}$ , although other acoustic measures are conceivable as a basis as well.

11. In the framework of null hypothesis significance testing (NHST), we compare the p values (for a definition, see Fahrmeir et al., 2016, p. 388) of fitted models with predefined alpha values (5%, 1%, and 0.1%) to reduce the risk of reporting false positive effects. One major problem of this procedure (comparing the *p* value) is that it operates on the assumption that the tested factor does not have an effect in the population underlying our sample. Conversely, it yields no numerical result about the case where the tested factor does have an effect in the population, which limits the procedure’s ability to decide whether the tested factor does or does not have an effect in the population. However, the procedure indeed has the advantage of being a standard procedure in the field of phonetics (and beyond). Pragmatically speaking, it also helps in spotting biased interpretations of raw data visualizations.

12. Normalization was applied to $closure_{norm}$ and $VOT_{norm}$ precisely to remove the effect of token-specific speech rate. It may seem counter-intuitive, then, to test for the effect of *speech rate* on these normalized measures. See Section 3.3.2 for an explanation.

13. To test for interactions in the random components, as suggested by one of the reviewers, we also ran the models in Equations 1 and 2 with a random component of (category*rate|speaker)+(speaker_group*rate|target_word) . As this yielded no substantial differences to the results of the original models, we will only describe the original ones in this paper (but see Section 3.3.3 for observations regarding between-speaker variation and see R code for results).

14. About 6% of combinations of speaker/word/condition have less than 5 usable repetitions and can, therefore, take other values than 0, 0.2, 0.4, and so on.
